# The Suppression of Vivisection

**Published:** 1889-06-29

**Authors:** 


### The Suppression of Vivisection.

The International Association for the Total Suppression of Vivisection held its annual meeting at the Westminster Palace Hotel on Thursday, the 20th inst. The meeting was largely composed of ladies, and the speakers included Lord De Ros, who occupied the chair, Miss Frances Power Cobbe, Sir William Plowden, M.P., Mr. Edward Berdoe, L.R.C.P., and several others. Besides the question of vivisection in general, two special points were under discussion—the proposed Leper Hospital and the proposed Pasteur Institute. Both were disapproved of, but not merely from the sentimental point of view. The really valid arguments against the scheme —valid in the eyes of thoughtful persons, whether or not they share the views of the opponents of vivisection, were those that have always been upheld in The Hospital—namely, that M. Pasteur's theory has not yet been proved to be perfectly sound, and M. Pasteur's results have not been yet so satisfactory, as to justify English money being spent on a Pasteur Institute, and the *prestige* of England's approval given to the inoculation theory. "The theory of inoculation," said Mr. Edward Berdoe at the meeting, "is that it is possible for the weak virus of a puppy to outrun and overcome the strong virus of a rabid dog." "We are asked to believe," says a writer in last week's Hospital, "that the weak virus, starting a day or two or several days later in the race, will outrun the stronger one, and seizing first upon the pabulum, for which the former is still more fiercely hungering, will destroy it before the latter can come up." The idea certainly does not commend itself to the common-sense of the average man; and without suggesting one iota of wilful cruelty or falsehood to scientific investigators, whom we know to be desirous simply of finding out the truth, we doubt greatly if the opinion of a scientist who is enamoured of a theory of his own invention is as reliable as that of the common-sense of the average man.

With regard to the Leper Hospital, everyone will feel that while it is fitting that a memorial should be raised to Father Damien, one of the noblest and most Christ-like souls of our age, and that this memorial should in some way benefit the sufferers, to whom he gave up his life, most people will feel also that a hospital where leprosy may be studied, and a cure devised, if cure there be, is a very fitting memorial; but is London the right place for that hospital? In London there is one leper, in all Britain there are at most, twenty. In India there are 250,000. At the meeting of the anti-vivisectionists, Prebendary Grier suggested that someone should study leprosy, not in London but in Molokai, among Father Damien's flock. The suggestion was a good one, but for us English, with our great Indian Empire, a hospital in India is more desirable.

Two aspects of vivisection were treated at the meeting; the sentimental and the scientific. Perhaps, however, as Miss Cobbe objected to the plea of sentimentality being used, it would be better to say that she appealed to the conscience, and Mr. Berdoe to the intellect, of their hearers. Miss Cobbe's appeal was both witty and powerful. Good comes out of evil, it is said—good to man comes out of the sufferings of the animals who are sacrificed to science. Some people say that good has come out of the Whitechapel horrors; but are we, on that account, asked Miss Cobbe, to tolerate, much less encourage, such crimes in our midst. "Shall we issue a license to Jack the Ripper, and have an inspector of murders?"

Mr. Berdoe, who is better known by his *nom de plume* of "Æsculapius Scalpel," the author of "St. Bernard's" and "Dying Scientifically," denied absolutely the good of vivisection. Experiments on animals always leave in doubt the question they are supposed to decide; and he hinted that in the action of drugs supplementary experiments were made on human beings. He expressed some doubt of the accuracy of the figures given in Mr. Erichsen's report, but explained them by the opinion expressed to him by a medical man who upheld vivisection. This gentleman said that he took pain in the physiological sense, meaning injury to a nerve, which excludes tetanus, spasms, and convulsions from "pain." On one point we must take issue with Mr. Berdoe. He quoted a remark made by Dr. Pringle, of the Indian Medical Service, who said that at the time the Punjaub was annexed the British Government gave orders that lepers should no longer be buried alive, "a command that did more credit to their philanthropy than to their knowledge of the laws of sanitation." Mr. Berdoe quoted at second-hand, and evidently thought that Dr. Pringle wished the burying of lepers to be continued. As it happens, we heard Dr. Pringle use those words, and we also heard the context. Dr. Pringle simply meant that the cessation of the burial of lepers had caused the disease to spread among the population. His desire is, we know, simply to see lepers segregated under proper supervision, in order that the contagion may not spread to others. We are sure that Mr. Berdoe would not willingly give a false impression of a brother physician.

